# Incidence of anastomotic leakage using powered circular staplers versus manual circular staplers for left colorectal anastomosis: a cost-effectiveness analysis

**DOI:** 10.1007/s10151-024-02936-0

**Published:** 2024-07-02

**Authors:** V. Pla-Martí, J. Martín-Arévalo, D. Moro-Valdezate, S. García-Botello, L. Pérez-Santiago, I. Barrachina-Martinez, S. González-de-Julián, D. Vivas-Consuelo, A. Espí-Macías

**Affiliations:** 1 Department of General and Digestive Surgery, Biomedical Research Institute INCLIVA, University Clinic Hospital of Valencia, Av. Blasco Ibáñez, 17, 46010 Valencia, Spain; 2 Department of Surgery, University of Valencia, Valencia, Spain; 3 Research Unit for Health Economics and Management, Universitat Politècnica de València, Camino de Vera S/N, 46022 Valencia, Spain

**Keywords:** Colorectal anastomosis, Anastomotic leak, Echelon circular stapler, Powered circular stapler, Cost-effectiveness

## Abstract

**Background:**

Colorectal anastomotic leakage causes severe consequences for patients and healthcare system as it will lead to increased consumption of hospital resources and costs. Technological improvements in anastomotic devices could reduce the incidence of leakage and its economic impact. The aim of the present study was to assess if the use of a new powered circular stapler is cost-effective.

**Method:**

This observational study included patients undergoing left-sided circular stapled colorectal anastomosis between January 2018 and December 2021. Propensity score matching was carried out to create two comparable groups depending on whether the anastomosis was performed using a manual or powered circular device. The rate of anastomotic leakage, its severity, the consumption of hospital resources, and its cost were the main outcome measures. A cost-effectiveness analysis comparing the powered circular stapler versus manual circular staplers was performed.

**Results:**

A total of 330 patients were included in the study, 165 in each group. Anastomotic leakage rates were significantly different (*p* = 0.012): 22 patients (13.3%) in the manual group versus 8 patients (4.8%) in the powered group. The effectiveness of the powered stapler and manual stapler was 98.27% and 93.69%, respectively. The average cost per patient in the powered group was €6238.38, compared with €9700.12 in the manual group. The incremental cost-effectiveness ratio was − €74,915.28 per patient without anastomotic complications.

**Conclusion:**

The incremental cost of powered circular stapler compared with manual devices was offset by the savings from lowered incidence and cost of management of anastomotic leaks.

## Introduction

Despite recent advances in preoperative patient preparation, enhanced recovery programs, and surgical techniques, anastomotic leakage (AL) remains a significant complication in patients undergoing colorectal surgery. Incidence varies on the basis of anatomical location, ranging from 1% to 23% in left colorectal anastomoses [1]. AL has multifactorial causes, and both patient- and surgical procedure-associated risk factors are well documented [2]. The consequences of AL are severe, leading to increased morbidity and mortality, prolonged postoperative hospital stays, and a heightened risk of re-interventions and permanent stomas [3]. Furthermore, AL adversely impacts long-term outcomes in oncological patients [4, 5].

The economic burden of AL is substantial due to its significant consumption of hospital resources [6]. Determining the precise cost of AL can be challenging, as it varies by country and healthcare system. Nonetheless, additional hospital expenditure can range from €14,711 to €71,978 [7–11]. To mitigate anastomotic complications and reduce the financial burden on hospitals, it is essential to conduct outcome audits and implement changes in clinical practices aimed at addressing modifiable risk factors [12–14]. Technological innovations in circular devices commonly used for colorectal anastomosis represent a modifiable factor and a potential target for risk reduction.

A new powered circular stapler, Echelon Circular™ (ECPS) [Ethicon, Somerville, NJ, USA], introduces technical innovation with the potential to enhance clinical outcomes. The powered firing process significantly reduces the force required for completing circular stapling and minimizes unintended movement along the anastomotic line. Atraumatic Gripping Surface Technology ensures tissue compression precisely where needed for staple placement. In conjunction with 3D Stapling Technology, this feature promotes more evenly distributed compression across the anastomosis, thereby enhancing intestinal microvascular flow [15]. Early clinical experiences with the ECPS have reported ease of use, enhanced safety, and reduced incidence of anastomotic complications compared to manual circular staplers (MCS) [16–19].

While the ECPS comes at a 35% higher cost than current MCS options, this cost difference could potentially be offset by reduced AL incidence and associated cost overruns in the hospital setting. The objective of the present study was to conduct a cost-effectiveness analysis to assess the use of ECPS versus traditional MCS for performing colorectal anastomosis.

## Methods

An observational study was conducted on patients undergoing left-sided circular stapled colorectal anastomosis between January 2018 and December 2021 at a tertiary public university hospital serving approximately 320,000 inhabitants. The study received approval from the hospital’s research ethics committee. All patients signed the institution informed consent for colorectal surgery. Inclusion criteria comprised patients over 18 years of age undergoing elective colorectal anastomosis with a circular stapling device, irrespective of anastomosis level or need for a diverting stoma, with a minimum follow-up period of 1 year. Cases involving a transanal total mesorectal excision approach were excluded. Patients were categorized into two groups based on whether the anastomosis was performed using MCS or ECPS. ECPS was introduced in our hospital in June 2019 and used preferentially unless otherwise unavailable. A retrospective analysis of data collected prospectively from the Coloproctology Unit database was conducted to assess differences in AL rates.

The perioperative patient management protocol adhered to ERAS principles and remained consistent throughout the study period, with all surgeries performed by the same group of six colorectal surgeons, with more than 10 years of experience, following the same surgical principles. In all cases, C-reactive protein (CRP) levels were assessed 72 h post surgery. Endorectal contrast-enhanced computed tomography (CT) was conducted if CRP values exceeded 200 mg/dl or there was a clinical suspicion of AL. For patients with defunctioning stomas, anastomosis evaluation occurred at the outpatient clinic via rectoscopy and contrast enema before ileostomy closure. AL was defined and graded according to International Study Group of Rectal Cancer criteria [20]. Grades A and B referred to cases managed with conservative treatment, while grade C indicated the need for surgical intervention.

Study variables included age, sex, Charlson comorbidity index, ASA (American Society of Anesthesiologists) score, neoadjuvant treatment, surgical approach, primary diagnosis, need for diverting stoma, the specific type of circular stapler used (MCS or ECPS), and surgical duration; outcome variables encompassed AL, postoperative complications categorized following the Clavien-Dindo classification, incisional surgical site infections, paralytic ileus, and the need for permanent colostomy or ileostomy.

### Statistical analysis

Descriptive statistical analysis was conducted using absolute and relative frequencies for qualitative variables, while quantitative variables were expressed as mean ± standard deviation or median (range) based on their distribution.

Between-group comparisons of patient demographics, diagnoses, and procedures were made using parametric (Student* t*) and non-parametric (Mann–Whitney* U*) tests, depending on the normality of the variable.

Propensity score matching was also carried out to form two comparable groups, one for each stapling device type utilized. A logistic regression algorithm was employed for matching, with a caliber of 0.2 and a group ratio of 1:1. The groups were matched for confounding variables including age, sex, Charlson index, diagnosis, and surgical procedure.

After two comparable groups were achieved, potential disparities in anastomotic complications were investigated using the chi-square test, and odds ratios were computed. Next, a cost-effectiveness analysis was conducted on the newly formed, matching-adjusted patient groups. Cost-related quantitative variables were standardized and normalized using the Z-score technique. Following this, potential differences in associated costs between study devices were assessed using parametric tests, specifically the Student* t* test.

A* p* value of ≤ 0.05 was considered statistically significant. R software (version 4.2.2) and the Amua and TreeAge applications were used for the analysis.

### Cost-effectiveness analysis

An economic evaluation comparing the costs and outcomes associated with two different circular stapler devices (ECPS and MCS) was carried out via cost-effectiveness analysis, a systematic approach for comparing two or more alternative procedures by assessing both costs and consequences (health outcomes).

A decision tree model was constructed for the two devices. The branches of this tree represent the management protocols employed by the Coloproctology Unit in cases of AL, drawing from our own experience and supported by recently published studies [21, 22].

Direct costs related to diagnostic and therapeutic procedures were computed for each patient on the basis of medical record review conducted by the primary authors of the study. Indirect costs such as sick leave, reduced productivity, and psychological damage were not included because of the challenges associated with quantifying them.

A database was created with a record for each patient’s direct costs, encompassing expenses associated with operating room, stapling device, surgery type, re-interventions, imaging diagnostics, laboratory tests, complications, and hospital and intensive care unit stays for each treatment. Follow-up visits to surgical and ostomy outpatient clinics were also factored in as costs, while pharmaceutical costs were excluded because of the inability to individually pinpoint them. The costs associated with each hospital resource were estimated in accordance with the tax law of our regional government for the year 2021 [23]. Effectiveness was measured on the basis of the total number of definitive stomas.

The economic evaluation was conducted using a cost-effectiveness analysis (CEA), which involved comparison of costs and outcomes.

Finally, the incremental cost-effectiveness ratio (ICER) was calculated by dividing incremental cost by incremental effectiveness, interpreted as the additional cost per patient without anastomotic complications.

$$\mathrm{ICER}= \frac{\mathrm{Delta Costs}}{\mathrm{Delta Effectiveness}}=\frac{\mathrm{ECPS Total costs - MCS Total cost}}{\mathrm{ECPS effectiveness - MCS Effectiveness}}$$

### Probabilistic sensitivity analysis

A Monte Carlo analysis was conducted as a sensitivity analysis to validate the robustness of the results. A total of 1000 simulations were executed using the Monte Carlo simulation method, and outcomes were illustrated on a cost-effectiveness plane.

## Results

**Fig. 1:**
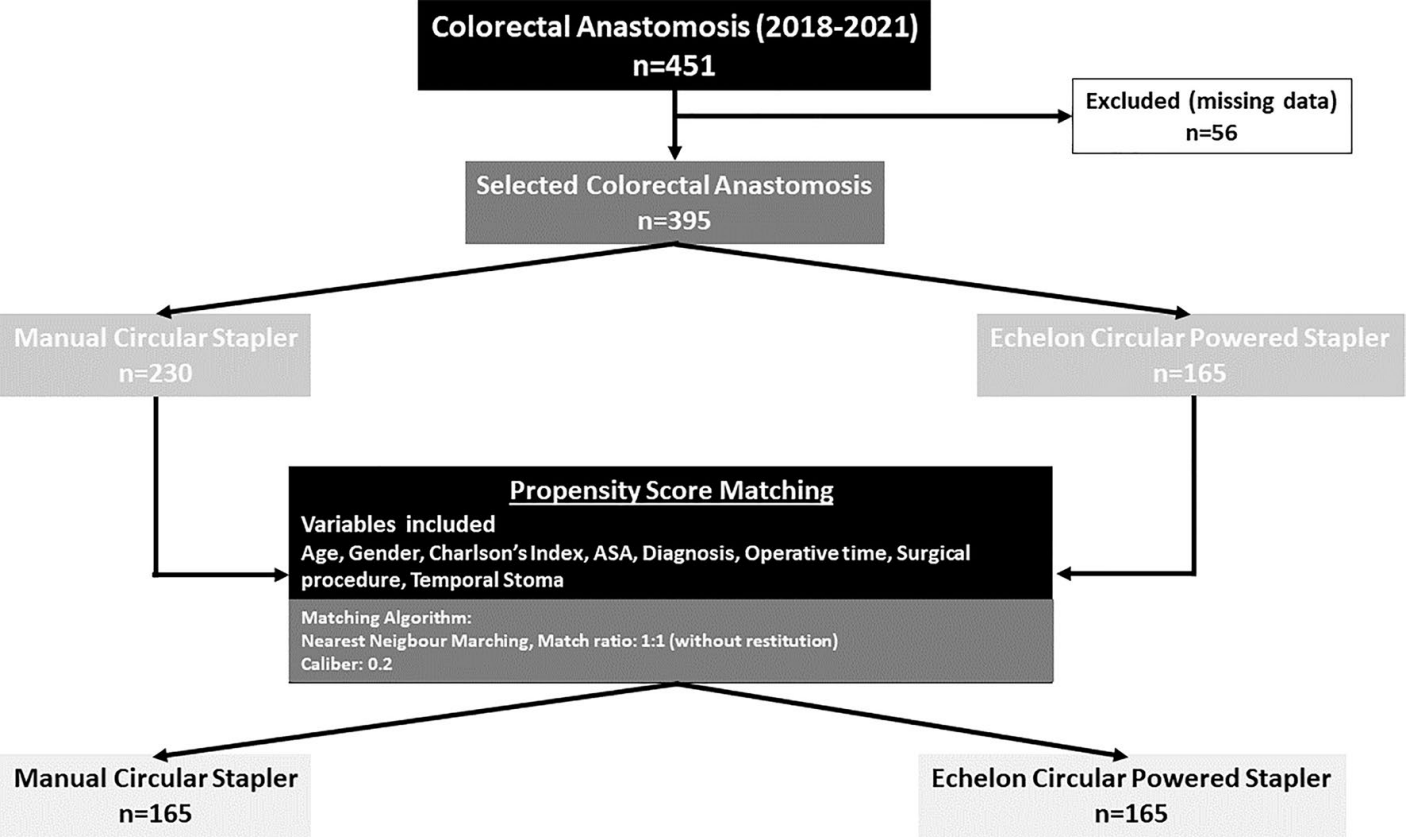
Flowchart of population selection and matching by propensity score. *ASA* American Society of Anesthesiologists

A total of 451 patients underwent colorectal anastomosis during the study period, 395 of whom met the inclusion criteria. Propensity score matching yielded two fully comparable groups, each consisting of 165 patients, categorized according to the type of circular stapling device used for colorectal anastomosis (Fig. 1).

No significant between-group differences were observed as regards demographic and pathological characteristics (Table 1).

**Table 1:** Description of the demographic and pathologic characteristics of the study group

	MCS *n* = 165	ECPS *n* = 165	*p* value
Sex (female)	64 (38.8)	64 (38.8)	1
Age (years)^a^	65 (67)	68 (60)	0.364
BMI (kg/m^2^)^a^	26.62 (22.25)	26.02 (34.37)	0.651
Charlson’s index^a^	4 (11)	5 (11)	0.361
ASA			0.390
I	6 (3.6)	3 (1.8)	
II	95 (57.6)	91 (55.2)	
III	63 (38.2)	67 (40.6)	
IV	1 (0.6)	4 (2.4)	
Diagnosis			0.159
Colon cancer	77 (46.7)	60 36.4)	
Rectal cancer			
Upper third (PME)	25 (15.2)	29 (17.6)	
Middle third (TME)	13 (7.9)	20 (12.1)	
Lower third (TME)	9 (5.5)	4 (2.4)	
Diverticular disease	18 (10.9)	23 (13.9)	
Hartmann’s reversal	13 (7.9)	22 (13.3)	
Miscellaneous	10 (6.1)	7 (4.2)	
Diverting stoma	17 (10.3)	17 (10.3)	1
Laparoscopic approach	108 (65.5)	103 (63.4)	
Conversion to open approach	10 (9.3)	11 (10.7)	0.820
Operative time^a^	150 (378)	165 (386)	0.072
Stapler firings^a^	2 (3)	1 (2)	0.673
Tumor stage			0.097
0	7 (5.9)	6 (5.4)	
I	29 (24.4)	25 (22.5)	
II	48 (40.3)	29 (26.1)	
III	27 (22.7)	38 (34.2)	
IV	8 (6.7)	13 (11.7)	
Preoperative radiotherapy	14 (8.5)	17 (10.3)	0.571
Anastomotic leakage	22 (13.3)	9 (4.8)	0.014
A + B	7 (4.2)	0	0.336
C	15 (9.1)	9 (4.8)	0.289
Definitive stoma	11 (6.7)	5 (3)	0.199
Postoperative complications			0.463
0	101 (61.2)	113 (68.5)	
I	10 (6.1)	9 (5.5)	
II	26 (15.8)	21 (12.7)	
IIIa	5 (3)	2 (1.2)	
IIIb	13 (7.9)	10 (6.1)	
IVa	2 (1.2)	4 (2.4)	
IVb	5 (3)	1 (0.6)	
V	3 (1.8)	5 (3)	
Incisional surgical site infection	13 (7.9)	14 (8.5)	1.00
Paralytic ileus	26 (15.8)	13 (7.9)	0.04
Length of stay (days)^a,b^	7 (269)	6 (95)	0.02

Values in parentheses are percentages unless otherwise indicated. “Miscellaneous” diagnosis includes deep pelvic endometriosis, sigmoid volvulus, and chronic constipation

*MCS* manual circular stapler,* ECPS* Echelon Circular powered stapler, *BMI* body mass index. *ASA* American Society of Anesthesiologists. *PME* partial mesorectal excision. *TME* total mesorrectal excision

^a^Median (range)

^b^Total length of stay, includes hospital readmissions

Regarding AL rates analysis, significant differences were identified between the two cohorts (*p* = 0.012). In the MCS group, 22 patients (13.3%) experienced AL, 15 (9.1%) of whom required surgical treatment (grade C), while 7 (4.2%) were managed conservatively (grades A and B). In the ECPS group, 8 patients (4.8%) had AL, all of them requiring surgical treatment (grade C). The odds ratio for AL with ECPS was 0.510 (95% CI 0.279–0.931), compared with 1.54 (95% CI 1.20–1.97) for MCS. The number of patients needed to be treated (NNT) with ECPS to prevent one AL was 12.

The total and average costs of hospital resources analyzed in the two groups are presented in Table 2. Only device-associated costs were higher in the ECPS group; all other hospital resources costs were higher in the MCS group. The average cost per patient in the ECPS group was €6238.38, compared with €9700.12 in the MCS group.

**Table 2:** Description of each of the resource costs consumed in the study group according to the circular stapling device used

	Price (€)	Total cost		Average cost	
		MCS *n* = 165	ECPS *n* = 165	MCS *N* = 165	ECPS *N* = 165
Blood test	16	15,648	11,140	94.84 ± 176.3	69.33 ± 93.97
Microbiological study	40	11,200	3280	67.88 ± 266.59	19.88 ± 80.44
Abdominal x-ray	25.67	8548.11	4235.55	51.81 ± 188.3	25.67 ± 112.68
Gastrografin enema	119	1309	952	7.93 ± 29.77	5.77 ± 25.64
Ultrasonography	65.98	5146.44	3101.06	31.19 ± 76.96	18.79 ± 61.74
CT scan	182.61	14,434.09	8587.37	87.50 ± 213.14	52.05 ± 170.98
Interventional radiology	1796.13	25,144	8980	152.39 ± 803.34	54.42 ± 367.01
Operative time (min)	15	213,159.86	199,362.97	1291.88 ± 466.80	1208 ± 466.80
Circular stapler	MCS: 425	75,737	93,457	459.01 ± 134.06	566.41 ± 85.31
	ECPS: 553				
Blood transfusion	94.86	13,185.54	3509.82	79.91 ± 566.84	21.27 ± 83.38
Length of stay in surgical intensive care unit (days)	1365.29	110,588.49	15,018.19	670.23 ± 5269.7	91.02 ± 854.66
Hospital length of stay (days)	341	680,977	492,745	4127.13 ± 6182.39	2986.33 ± 3308.29
Medical consultation	40.2	26,571.3	25,686.8	161.04 ± 54.64	155.68 ± 48.66
Stomatherapy consultation	30.3	7919.2	4605.6	32.51 ± 76.45	27.91 ± 76.45
Ostomy devices^a^	200	92,400	29,800	1013.33 ± 6891.57	180.61 ± 529.25
		1,301,967.49	904,461.36	9700.12 ± 19,446.45	6238.38 ± 5649.2

All data in euros unless otherwise indicated

*MCS* manual circular stapler,* ECPS* Echelon Circular powered stapler

^a^Estimated cost per month provided by the stomatherapy unit

Upon scaling and normalization of cost variables, significant between-group differences were identified in the mean costs of microbiological studies (*t* = − 2.21, *p* = 0.03), simple radiological studies (*t* = − 1.43, *p* = 0.05), specialized medical consultations (*t* = − 0.94, *p* = 0.05), total days of hospitalization (*t* = − 2.09, *p* = 0.04), ostomy devices (*t* = − 2.78, *p* = 0.01), staplers (*t* = 8.68, *p* < 0.01), and total costs (*t* = − 2.15, *p* = 0.03). No statistically significant differences were observed in the other costs studied.

**Fig. 2:**
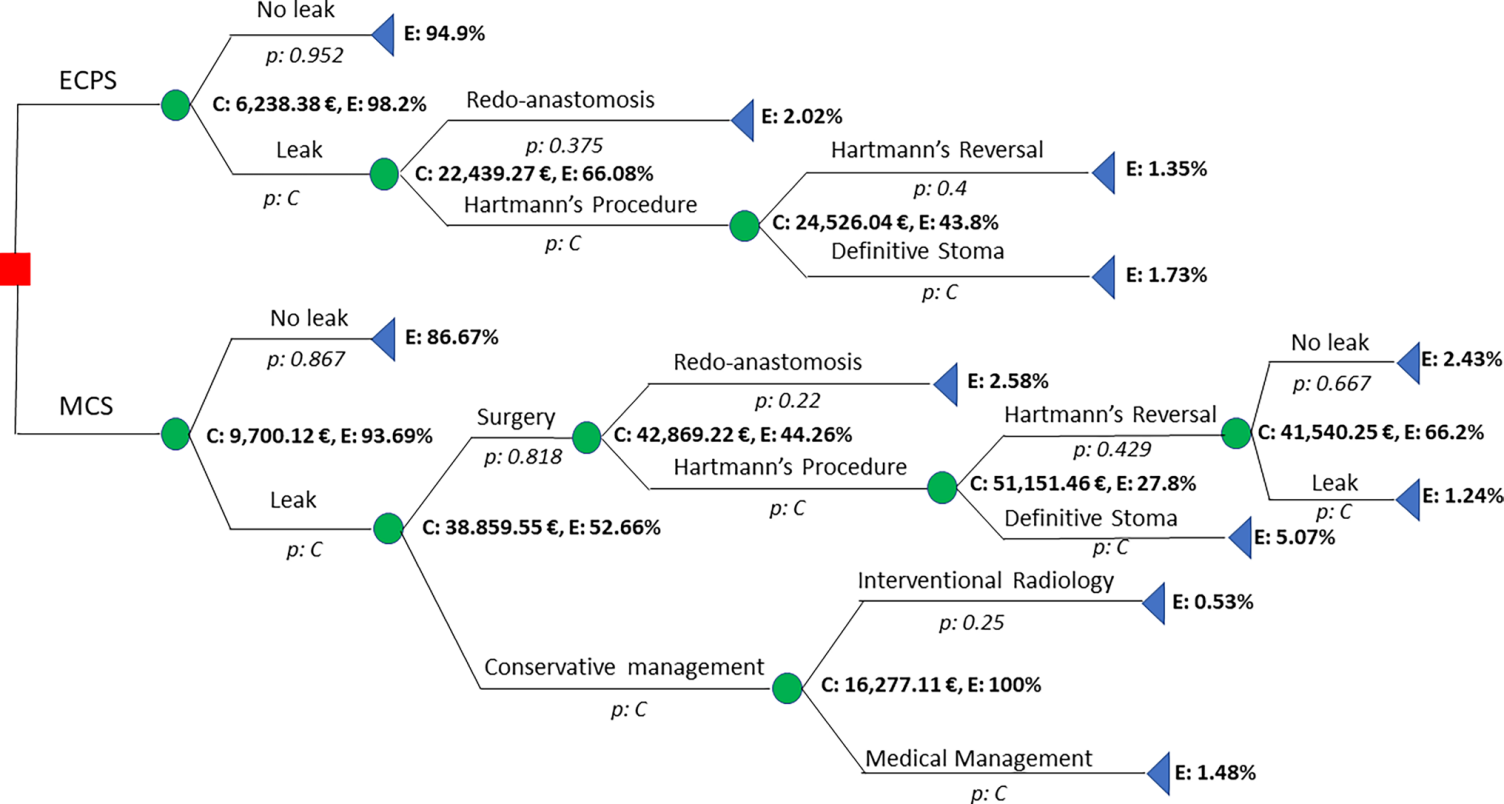
Decision tree and cost-effectiveness analysis regarding the utilization of the MCS and ECPS in relation to anastomotic leakage and the necessity for a definitive stoma. *ECPS* Echelon Circular powered stapler, *MCS* manual circular stapler, *p* probability, *C* complementary probability, *E* effectiveness (definitive stoma ratio)

**Fig. 3:**
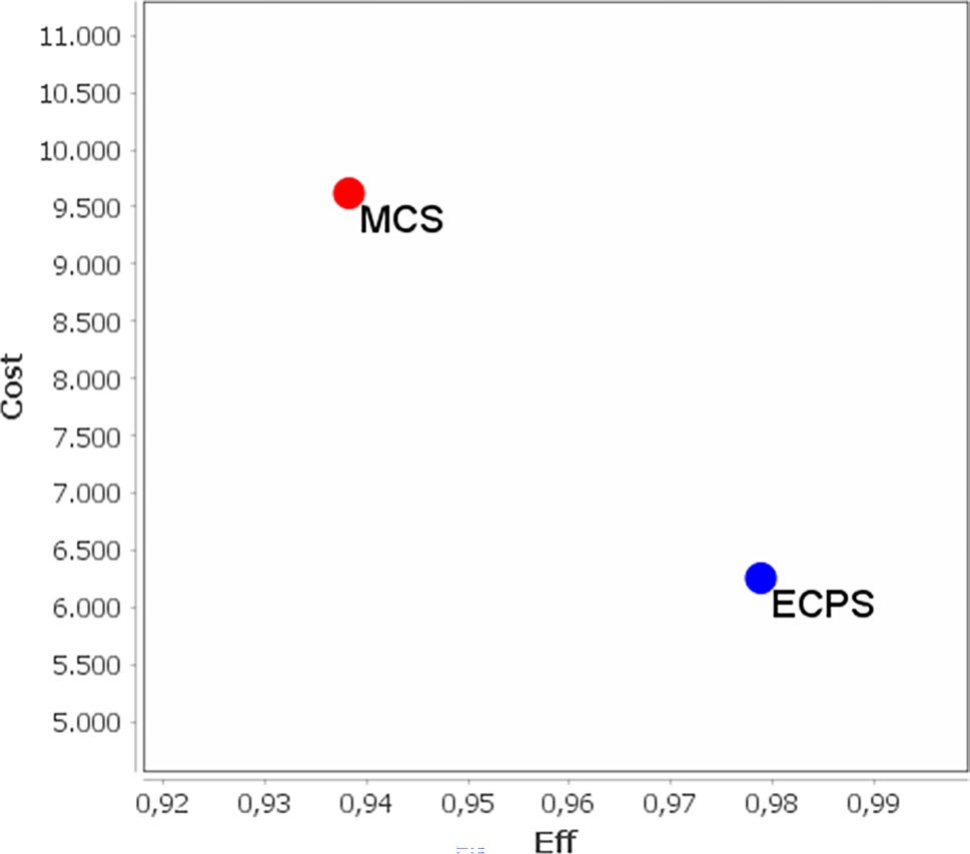
Cost-effectiveness ratio analysis of the manual circular stapler (MCS) and Echelon Circular powered stapler (ECPS) in relation to outcome variables

**Fig. 4:**
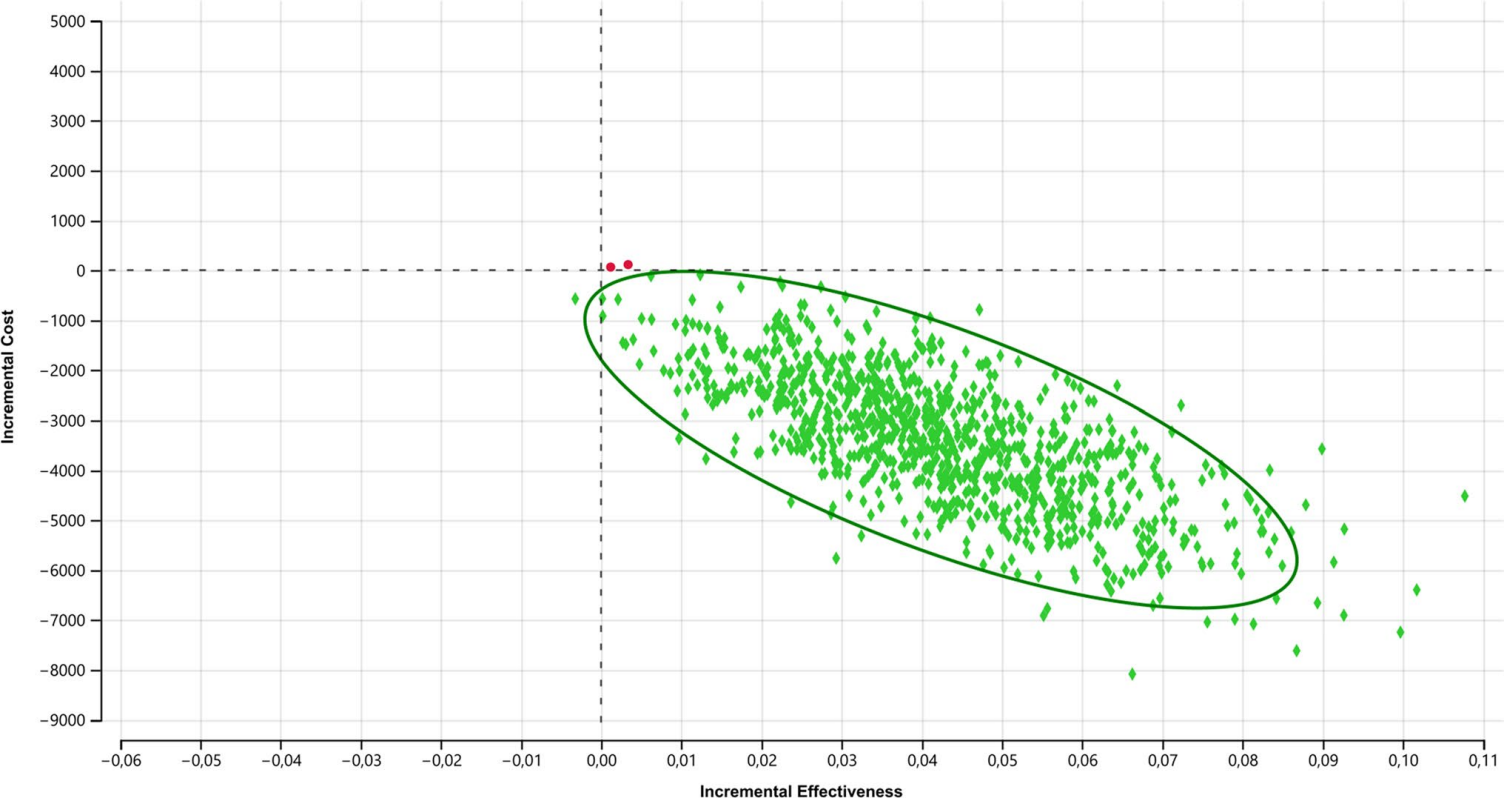
Monte Carlo simulation of decision tree model with 1000 simulations

The average hospital cost per patient who experienced AL was €30,649. Decision tree analysis (Fig. 2) revealed an efficacy of 98.27% for ECPS treatment, compared with 93.69% for the MCS group. ECPS demonstrated a superior cost-effectiveness ratio to MCS (Fig. 3). The ICER was − €74,915.28. In the Monte Carlo simulation (Fig. 4), nearly 100% of points fell within the second quadrant of the cost-effectiveness plane, signifying that ECPS is a dominant and cost-effective alternative compared with MCS.

## Discussion

For new health technologies to replace existing ones, they must not only provide clinical benefits but also demonstrate cost-effectiveness. Our initial experience with the new ECPS supported the former criterion, showing a decrease in AL rates compared to conventional MCS (1.7% vs. 11.8%) [17]. In the present study, we expanded the scope to include patients with anastomoses located less than 5 cm from the anal verge, those who underwent preoperative radiotherapy, and those with diverting ileostomy, a group excluded in our previous study. As anticipated, the AL rate was higher with the inclusion of higher-risk patients, although significant between-group differences remained (13.3% in the MCS group vs. 4.8% in the ECPS group). In addition to clinical benefits observed, this reduction in AL incidence has a substantial economic impact by lowering hospital costs. Despite the higher price of ECPS than MCS (a difference of €128), the average cost per patient was €3469 lower in the ECPS than in the MCS group.

Other studies have also demonstrated clinical advantages associated with ECPS. In a prospective multicenter single-arm study involving 12 centers in Europe and the USA, which included 168 patients undergoing colorectal anastomosis using the new ECPS, the AL rate was 1.8% [18]. Subsequently, a retrospective, matching-adjusted indirect study compared this ECPS patient cohort with a control group that had undergone colorectal anastomosis with conventional MCS, obtained from a national database in the USA. The AL rate was significantly lower in the ECPS group than the conventional MCS group (1.8% vs. 6.9%, *p* < 0.001) [19]. Using clinical data from this study, Pollack et al. assessed the economic impact of using ECPS compared to MCS. They developed a US hospital-based budget impact model analyzing total costs, average length of stay, proportion of patients with non-home discharge, and reasons for readmission. The reduction in AL with ECPS resulted in estimated annual savings of $53,987 assuming 100 procedures per year with each type of circular device, despite the higher cost of this device compared to MCS [24].

To the best of our knowledge, this is the first study to analyze the impact on using the new ECPS on colorectal AL reduction compared to the results obtained with MCS, in the same center, by the same group of surgeons, with no modification of perioperative protocols during the period, and with the type of circular stapler as the only change introduced. A further strength of our study is the analysis of economic impact considering the hospital resources actually used by each patient and their official cost in our setting.

The consequences of AL are undoubtedly of grave concern for patients, but it is equally crucial to consider the economic implications for the healthcare system. AL typically results in an increased burden on hospital resources, leading to a substantial economic impact. An Australian study examined hospital resources used for patients with AL after colorectal cancer resection in a cohort of 1228 patients. Treating the 41 patients who experienced leakage (AL rate of 3.8%) resulted in the following resource allocation: 92 days in intensive care, 129 days of total parenteral nutrition, 69 days of enteral feeding, 41 days on ventilation, and a median postoperative hospital stay of 28 days (range 11–104). These patients required 24 reoperations and 2273 separate medical consultations or additional services [6]. Although the cost of AL was not directly analyzed in the study, this significant increase in hospital resource consumption would evidently have substantial economic repercussions.

The financial implications of AL can vary depending on each country’s healthcare system. In many high-income countries, the diagnosis-related group (DRG) payment system is commonly used for hospital care reimbursement. Patients in the same DRG are expected to follow a similar clinical course, which should result in similar hospital costs. Surgical complications, including AL, are considered in DRG assignment and may contribute to higher reimbursement. However, AL often leads to substantially higher costs that may not be adequately covered by DRG reimbursement [25]. In a Swiss retrospective study, La Regina et al. compared resource use and DRG reimbursement between patients undergoing uncomplicated colorectal resection and those with AL. The cost for uncomplicated cases was €17,647, while patients with AL incurred costs of €71,978 (*p* < 0.01). The increase in costs was not fully compensated by the new complication-related DRG reimbursement, resulting in an average benefit per patient in the uncomplicated group of €542, while the AL group incurred an average loss of €12,181 per patient [11]. Similarly, an Italian retrospective study found that the mean adjusted hospital cost was 108% higher for patients with AL after colorectal surgery (€14,711 vs. €7089). The average DRG reimbursement for patients with AL covered only 86% of hospitalization costs, resulting in an average loss per patient with AL of €2041 [7]. In Spain, a recent study estimated the additional cost of diagnosing and treating AL following colorectal cancer surgery to be €38,819 for patients with colon cancer and €32,599 for patients with rectal cancer [26].

The economic impact of AL can be viewed from both the hospital’s and the payer’s perspective. Hospitals may not be fully compensated by payer reimbursements for the actual cost of resource consumption, and payers may also bear additional expenses, such as readmissions or stoma care [27]. Therefore, reducing AL incidence is essential not only for patient well-being but also to preserve the sustainability of the public healthcare system, especially in settings with fixed annual budgets such as our hospital.

Our study has several limitations that warrant consideration. Firstly, it is a retrospective observational study conducted at a single medical center, and patients in each group were treated at different time periods to allow for ECPS selection as the device under study. To mitigate potential bias, we carefully restricted the study period, ensuring uniformity in perioperative protocols and consistent surgical procedures performed by a cohesive team of colorectal surgeons, each boasting over a decade of experience. Moreover, we employed propensity score matching to create comparable patient groups.

Secondly, owing to characteristics of our healthcare system and the retrospective design of the study, detailed information on pharmaceutical expenses for individual patients was not accessible, thus ruling out the possibility of including these costs in our hospital resources analysis. While this omission may have resulted in slight underestimation of the actual costs, it is unlikely to significantly affect the differences observed between the two groups. Finally, other resource use-related factors not considered in our analysis could potentially have influenced the results. In addition, this study was not designed to assess risk factors for AL and although an attempt was made to make the groups homogeneous in this regard, some unconsidered factors could affect the results.

Despite these limitations, our study highlights the positive impact of introducing ECPS into clinical practice, particularly in reducing AL rates. While a cost differential between ECPS and MCS exists, the hospital savings from the reduction in AL cases more than compensate for this difference.

## Conclusion

Incorporating ECPS into our clinical practice has yielded favorable results by significantly reducing the AL rates. While the initial cost investment in ECPS exceeds that of MCS, the subsequent reduction in AL cases results in substantial savings for the hospital. ECPS demonstrates superior effectiveness and a lower cost per patient than MCS.

## Data Availability

The datasets generated during and/or analyzed during the current study are available from the corresponding author on reasonable request.
